# Fruit and Vegetable Wastes as Alternative Animal Feed for Small‐Scale Horticultural Farmers: Case of Gamo Zone, Southern Ethiopia

**DOI:** 10.1002/vms3.70349

**Published:** 2025-04-14

**Authors:** Mitiku Yohannes, Yisehak Kechero, Yilkal Tadele

**Affiliations:** ^1^ Department of Animal Science College of Agricultural Sciences Arba Minch University Arba Minch Ethiopia

**Keywords:** avocado by‐products, banana by‐products, cabbage leaf, cassava by‐product, fruits and vegetable waste/by‐product, mango by‐product, potato peel

## Abstract

In many sub‐Saharan African countries, the demand, supply and quality of farm animal feed are not proportional. As a result, searching for low‐cost, cheaply available feed biomass has been considered an alternative measure and solution. The purpose of the study was to assess feed resources, the potential of fruit and vegetable by‐products as alternative feeds and practical utilization of livestock in three selected districts of Gamo Zone, Southern Ethiopia. Data were collected through household interviews, focus group discussions and available biomass estimation carried out on fruit and vegetable‐based farms using weighing balances. The results indicated that livestock holding status, land size and cultivated crops varied (*p* < 0.05) across three agro‐ecological zones. From a hectare of land with varying yields of dry matter (DM), 24.14 MT of banana by‐products, 1.76 MT of mango by‐products and 0.72 MT of avocado by‐products were obtained. A hectare of vegetable and tuber croplands produced 22.7 MT of sweet potato by‐products, 1.41 MT of cassava by‐products, 1.12 MT of potato peels and 0.55 MT of cabbage leaf fresh biomass. As a result, diverse agro‐ecologies and seasons can make use of these biomasses as a supplement and replacement for conventional feed sources.

## Graphical abstract

Major feed and crop residue contributions to livestock feed identified in the area.

Production and potential of fruit‐based by‐product as alternative feed resources identified in the area.

Potential of tubers and vegetable by‐product and practical utilization as animal feed verified in the area.

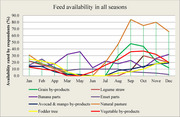

## 1 Introduction

Livestock feed resources in Ethiopia are classified as natural pasture, crop residue, improved pasture and forages, agro‐industrial by‐products and other by‐products such as vegetable and fruit refuse, of which the first two contribute the largest share (Fentahun et al. 2020; Gebremariam and Belay 2023). Natural pastures are generally poor in quality, and their productivity and volume of biomass supply are seasonal, particularly limited during the dry season.

The grazing land management and feed quality improvement activities are rarely adopted in the country. Thus, the existing grazing lands are poor in terms of species diversity, low in biomass yield and poor in nutrition. Crop residues are important sources of feed in the mixed crop–livestock production systems of Ethiopia (Abera et al. 2014; Duncan et al. 2016). However, crop residues have nutritional limitations that may obstruct their efficient utilization for livestock feeding. Commercial concentrate mixtures and other agro‐industrial by‐products are good sources of supplements; however, they are not easily affordable and accessible to many smallholders due to inadequacy of agro‐processing industries in the country. Further, lack of availability of high‐quality commercial feed is also cited as a constraint for livestock productivity under resource‐poor livestock production systems in combination with the unaffordable feed costs, in case farmers are resorting to whatever resource is available to feed their animals (Yisehak and Janssens 2014; Mutwedu et al. 2022).

The current contribution of livestock to producers and to the national economy is by far the smallest. Among the many factors that could explain the disproportionate role of the livestock sector is mainly inadequacy and poor quality of available feed resources (Balehegn et al. 2021). Smallholders in mixed crop–livestock production systems, keep some types of livestock in combination with crop production. Nevertheless, according to the study findings of Mekuria and Kindu (2018), cropland increased by 16%, whereas grazing lands decreased by 52% from 1984 to 2016 in similar mixed farming systems of the Central Ethiopian highlands.

The development of cash crops like bananas, mangos, avocados and other vegetables in the research area currently has resulted in a shortage of grazing lands and a limitation of grain‐ and cereal‐based crop residues. On the other hand, the huge feed biomass generated as a by‐product from the fruit and vegetable farms during harvesting and collection seasons is commonly discarded as waste, damped in open fields. Searching for feed substitutes within declined trends of fodder production and an increase in production input opens door for the efficient disposal of fruit and vegetable wastes by offering the best way to develop feed resources (Dida et al. 2023).

For reducing feed cost and increasing farm animal productivity, it is important to exploit locally available feed sources and make them available to users (Dida et al. 2023). Thus, understanding and identifying cheaply available biomasses could be an imperative and scientific approach to improve livestock production in the area. Therefore, the current study was planned to assess the livestock feed potentials of fruit and vegetable wastes across different altitudes and seasons and their feeding practices in Gamo zone of Southern Ethiopia.

## 2 Materials and Methods

### 2.1 The Study Area

The study was conducted in Gamo Zone, Southern Ethiopia. Located at 5°57–6°71′ N and 36°37′–37°98′ E, the general elevation of the area ranges from 680 to 3880 m above sea level. The area has three distinct agro‐ecological zones, that is, Dega (high altitude), Woyina‐Dega (mid‐altitude) and Kola (low altitude), respectively. The specific study areas included in the Gamo zone are Arba Minch Zuria, Mirab Abaya and Dera Malo districts.

### 2.2 Sampling Procedures and Sample Size

Multistage sampling procedure was employed to select the study units of this research. In the first stage, three districts, namely, Mirab Abaya, Arba Minch zuria and Dera Malo, were selected purposively for being known for their livestock production potential and representing the mixed crop‐livestock production, cropping patterns and agro‐ecological settings of Gamo zone. Each district was then sub‐stratified into three agro‐ecological zones: high, mid and low altitudes, respectively. Nine villages (lower administrative sub‐units or wards) were selected following a purposive sampling technique: three from each district. The respondents were selected from each *kebele* by simple random sampling technique by considering households that engaged in fruit and vegetable production and possessed livestock production. The selection of respondents for FGDs was done purposefully by *kebele* leaders and development agents of respective *kebeles*, and about 10 respondents from each district, a total of 90 farmers, were selected. The total sample size for the household interview was determined by using a probability proportional to the size‐sampling technique designed by Cochran (1977). The margin of sampling error was calculated for a 99% confidence interval with 10% fraction and large indefinite population. Accordingly, a representative sample for proportions in large populations was calculated as follows:

$$n_{0}=\frac{Z^{2}\;\times \;p\;\times \;\left(1\;-\;p\right)}{e^{2}}$$

where *n*_0_ is the desired sample size (for large or infinite populations); *Z* is the *Z* value found in the *Z* table at a given confidence level (e.g., it is 2.58 for a 99% confidence level); *p* = 0.1 (estimated proportion of an attribute that is present in the population, i.e., 10%); *q* = 1 − *p*, that is, (0.9); and *e* ^2^ is the acceptable sampling error (0.05).

Therefore, *n*_0_ is equivalent to 240 respondent farmers. Finite population correction for proportions was calculated as follows:

$$n=\frac{n_{0}}{1+\frac{\left(n_{0}-1\right)}{N}}$$

where *n*_0_ is the initial sample size; *n* is the adjusted sample size; and *N* is the population size.

The Cochran formula for finite population correction for proportions, the second formula, *n* was used. *N* is the total number of the population experiencing all fruit, vegetable and livestock farming (i.e., especially model horticulture producer household heads as a target population = 12,000 for three districts). The target population related to fruit and vegetable farming communities was obtained from the respective district offices. A landless individual was not included in the study. Therefore, *n* is the required sample size, which is equivalent to 235. For the representation, about 45% (=106), 40% (*n* = 94) and 15% (*n* = 35) of respondents were sampled from lowland, mid‐land and highland altitudes respectively, based on proportions of areas altitudinal characteristics obtained from district agricultural offices. The biomass measurements for all fruit and vegetable by‐products were carried out on a hectare basis of land size after harvesting of fruit and vegetable products for consumption or during supplying to market.

### 2.3 Data Collection Methods

The qualitative and quantitative data were collected through face‐to‐face individual household head interviews using a semi‐structured questionnaire with open‐ and closed‐ended questions. The FGDs, field observations and biomass estimations were carried out to assess existing conventional feed resources, fruit‐ and vegetable‐based by‐products, their availability, trends and variability in the area. Moreover, utilization practices and feed management aspects were assessed in all agro‐ecological zones and dry and wet seasons of the study area through questionnaires and checklists developed for FGDs. In addition to this, measurements and quantity estimations of available fruit and vegetable production on a hectare basis and their by‐products that were discarded or provided to animals following the questionnaire for total production aspects and the sample biomass were measured by using a weighing balance. The fresh/dry weight relationships were calculated for each sample to convert the total fresh weight (TFW) of each component of the biomass recorded in the field into dry weight following standard biomass measurement procedures.

The dry matter (DM) yield of each species was determined in an oven (65°C for 72 h) (AOAC 2019). On the basis of the DM weights obtained from sample sites, the percent composition of each species of biomass per total production size was calculated, and the total biomass production capacities of the area were recorded according to Tothill et al. (1978):

i. TDW of individual species = TFW of a species × SDW of species × SFW of a species.
ii. % Composition of each species at a site = TSFW of a species per GTFW of all the species

where TFW is the total fresh weight of individual species, SFW is sub‐sample fresh weight, SDW is sub‐sample dry weight, TDW is total dry weight and GTFW is the total fresh weight of all species.

For parameters required for ranking, an index value was calculated as the sum of the weighted numbers of responses for the criterion to provide an overall ranking of qualitative data according to the formula used by Sisay et al. (2021):

Index = sum of (3 × number of households ranked 1st + 2 × number of households ranked 2nd + 1 × number of households ranked 3rd) given for an individual reason, criteria or preference divided by the sum of (3 × number of households ranked 1st + 2 × number of households ranked 2nd + 1 × number of households ranked 3rd) for overall reasons, criteria or preference.

The variable with the highest index value was the most economically important. The indices were calculated as follows:

$$\begin{array}{ccc} & & \mathrm{Index}=\\ & & \;\frac{\sum \left(Rn\times C1+Rn\times C2\text{…}\text{…}.+R1\times Cn\right)\;\mathrm{for}\mathrm{individual}\mathrm{variable}}{\sum \left(Rn\times C1+Rn\times C2\text{…}\text{…}.+R1\times Cn\right)\;\mathrm{for}\mathrm{all}\mathrm{variable}} \end{array}$$

where *Rn* is the last rank; *Cn* is the % of respondents in the last rank; *C*1 is the % of respondents ranked 1st.

### 2.4 Statistical Analysis

The collected data from the household survey were analysed by using the Statistical Package for Social Sciences (SPSS version 26). The statistical variations for categorical data were tested by means of cross‐tabs, and the significance level was declared at (*p* < 0.05). The means of quantitative data among agro‐ecologies and between seasons were computed independently by using one‐way analysis of variance. The statistical model used for analysing data on available feed resources and utilization practices was *Y*_ijk_ = *μ* + *β*_j_ + *ε*_ijk_, where *Y*_ij_ is the total observation due to *i* and *j* factors; *μ* is the overall mean; *α_i_* is the *i*th effect of location (agro‐ecologies); *β*_j_ is the *j*th effect of production season (dry and wet); *ε*_ij_is the random error.

## 3 Results

### 3.1 Demographic Characteristics of Respondents

The demographic data of respondent farmers are displayed in Table 1. The majority of household heads included in this study were male, whereas a small percentage (4.5%) were represented by females. The majority of respondents in the current survey were between the ages of 36 and 45, whereas respondent with ages less than 25 and above 56 accounted for a small percentage (1.8%) and (3.5%), respectively.

**TABLE 1:** Demographic and education status of the respondents in the study area (%).

Variable	Agro‐ecology (%)			Over all	*p* value
	Lowland	Mid‐land	Highland		
Gender					
Male	92.5	96.8	94.3	95.5	0.28
Female	7.5	3.2	5.7	4.5	
Age category (years)					
<25	1.8	1.0	2.8	1.8	
26–35	3.7	3.2	5.7	4.2	
36–45	45.3	48.9	51.4	48.5	
46–55	47.2	43.6	34.3	41.7	0.24
>56	1.8	3.2	5.7	3.5	
Family size (%)					
1–3	14.2	12.8	14.3	13.7	0.34
4–7	66.0	69.2	60	65.1	
8–11	16.1	15.9	22.8	18.3	
≥12	3.8	2.1	2.8	2.9	
Educational status (%)					
Illiterate	51.8	55.3	48.5	51.8	
Elementary level	40.5	41.4	42.8	41.5	0.433
High school level	6.6	3.2	8.5	6.1	
Certificate and above	0.02	0.00	0.00	0.006	

*Note*: ‘%’ denotes percentage.

The majority of respondents family sizes, as obtained from the current study, are (4–7), followed by (8–11), (1–3) and (≥12), respectively. The educational status of respondents revealed that the majority were illiterate (51.8%), followed by elementary (41.5%), high school level (6.1%) and certificate level (0.006%), respectively.

### 3.2 Land Size and Proportional Allocation of Land for Agricultural Activities

The total land size and proportion of land to different agricultural productions are presented in Table 2. The result indicated that the plot of cropland allocation to a particular crop type and proportion of farmers who have grown different crops during the cropping seasons varied (*p* < 0.05) in all agro‐ecological zones. The wider parcel of land belongs to lowland, followed by mid‐land and highland, from which the higher size is occupied by fruit and vegetable, cereal and grain crops at lowland, mid‐land and highland agro‐ecologies, respectively.

**TABLE 2:** Land holdings (hectare basis) and their allocation to various agricultural productions.

	Agro‐ecology (mean)					
Land size (hectare)	Lowland	Mid‐land	Highland	Overall mean	S.E.M.	*p* value
Total land area	1.84^a^	1.43^b^	1.12^c^	1.46	0.021	0.042
Cereal and grain crops land	0.35^c^	1.03^a^	0.76^b^	0.71	0.033	<0.001
Fruit and vegetable land	1.38^a^	0.14^b^	0.12^b^	0.55	0.021	0.005
Grazing land	0.03^b^	0.12^a^	0.11^a^	0.08	0.052	<0.001
Trees and shrubs land	0.06^b^	0.11^a^	0.09^a^	0.08	0.014	0.009
Others	0.02	0.03	0.04	0.03	0.062	0.401

*Note*: Means with different superscripts (a, b, c) in the same row are varied significantly (*p* < 0.05).

Abbreviation: S.E.M., standard error of mean.

### 3.3 Livestock Holding Status of the Respondents

Livestock holding status and the proportion of livestock species of the respondents are presented in Table 3. Cattle and poultry populations were equally distributed across agro‐ecological zones and did not vary statistically (*p* > 0.05).

**TABLE 3:** Livestock holding status of the respondents.

	Agro‐ecology (mean)					
Species	Lowland	Mid‐land	Highland	Overall mean	S.E.M.	*p* value
Cattle	2.14	2.35	2.35	2.28	0.181	0.166
Sheep	0.50^b^	1.65^a^	2.15^a^	1.43	0.213	<0.001
Goat	2.29^a^	1.12^b^	0.48^c^	1.29	0.222	<0.001
Equines	0.15^b^	0.38^a^	0.81^a^	0.44	0.061	0.022
Poultry	5.10	4.98	4.80	4.96	0.394	0.445

*Note*: Means with different superscripts (a, b, c) in the same row are varied significantly (*p* < 0.05).

Abbreviation: S.E.M., standard error of mean.

Sheep and equine populations owned by respondents varied with agro‐ecological zones, with higher numbers (*p* < 0.05) at highland and mid‐land and lower numbers at lowland areas. The goat distribution was vice versa to sheep and equine distribution; the higher (*p* < 0.05) was for lowlands, and the lower was for highland and mid‐land agro‐ecological zones, respectively.

### 3.4 Major Feed and Crop Residue Contributions to Livestock Feed

Available feed resources, including crop residues, were prioritized by respondents in each agro‐ecological zone based on the perceived contributions presented in Table 4. The result indicated that natural pasture ranked 1st irrespective of agro‐ecological zones, followed by grain‐based crop residues, which ranked 2nd for all agro‐ecological zones, whereas maize‐stover is the most noticeable grain‐based crop residue, with lowland tef straw, barley straw and sorghum stover in mid‐land and wheat and barley straw as the main sources of grain‐based crop by‐products in highland areas of the study districts.

**TABLE 4:** Livestock feed types, including fruit and vegetable wastes, prioritized by respondents.

Feed type	Agro‐ecologies, index and rank							
	Lowland		Mid‐land			Highland		
	Index	Rank	Feed type	Index	Rank	Feed type	Index	Rank
Natural pasture	0.19	1	Natural pasture	0.24	1	Natural pasture	0.23	1
Grain‐based crop residues	0.16	2	Grain crop residues	0.22	2	Grain crop residues	0.20	2
Banana parts	0.13	3	Enset parts	0.12	3	Enset parts	0.13	3
Weeds and shrubs	0.09	6	Legume straw	0.08	6	Tuber crop wastes	0.10	5
Mango/Avocado wastes	0.11	4	Tuber crop wastes	0.05	7	Legume haulms	0.06	8
Fodder trees	0.10	5	Vegetable wastes	0.11	4	Vegetable wastes	0.11	4
Tuber crop wastes	0.08	8	Weeds/Shrubs	0.10	5	Weeds and shrubs	0.08	7
Vegetable wastes	0.09	7	Fodder trees	0.08	6	Fodder trees	0.09	6

*Note*: Index = sum of (4 × cn ranked 1st) + (3 × cn ranked 2nd) + (2 × cn ranked 3rd) + (1 × cn ranked 4th)/the sum of (4 × cn ranked 1st) + (3 × cn ranked 2nd) + (2 × cn ranked 3rd) + (1 × cn ranked 4th) for overall preference.

In lowland areas, among non‐conventional feeds potentially used as alternative feed during dry seasons, banana by‐products, mango and avocado seeds, fodder tree leaves and weeds and shrubs ranked 3rd, 4th, 5th and 6th, respectively, but in mid‐ and highland areas, *Enset* (*ventricosum)* by‐products and vegetable wastes and tuber crop wastes together with haulms and straws from legumes (haricot beans, field peas and beans) were considerably used and ranked 3rd, 4th, 5th and 6th, respectively.

### 3.5 Trends and Availability of Different Feed Resources With Seasons

**FIGURE 1:**
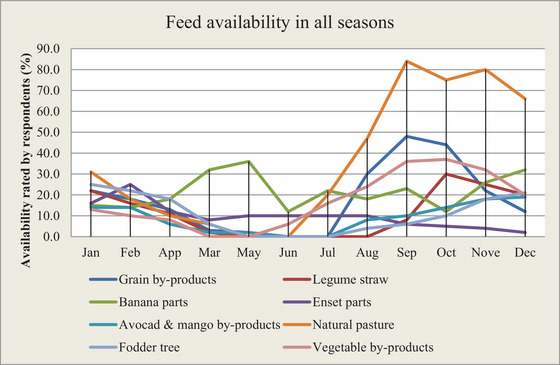
Availability of different feed resources in all seasons.

Major feed resources and their availability in each season are illustrated in Figure 1. The result showed that natural pasture, *Enset* (*ventricosum)* parts, vegetable and banana by‐products were widely used throughout the year in the study districts. The majority of crop residues (grain‐ and legume‐based straws) were available in dry seasons (October to January) in the study area. The fluctuation and variability of availability between seasons of major feed resources is mainly associated with the rainfall distribution and cropping pattern of the area.

Natural pasture resources contribute throughout the year but are abundantly available during the main rainy seasons (May to September), commonly the peak crop cultivation seasons in the area.

### 3.6 Annual Production Potential of Fruit‐Based Wastes as Feed

The potential of estimated biomass and feed resources generated from fruit‐based farms are presented in Table 5. The amount of biomass recorded and estimated for each fruit per hectare of land was indicated as 24.14 MT of (leaf, discarded fruit, pseudo stem and peel) biomasses from banana farms, with varied (*p* < 0.05) DM yields for both wet and dry seasons. Except for banana peels, all other banana by‐products, such as banana fruit, leaves and discarded banana fruit per hectare of land recorded from year‐round banana production, significantly varied (*p* < 0.05) between seasons. The records of by‐products and leftovers obtained from the mango farm, represented by 0.83 MT of mangos (22.73% of total production size per annum) and 0.93 MT of seed kernels, a total of 1.76 MT of fresh biomass were varied (*p* < 0.05) between seasons with diverse (%DM) yields. Similarly, avocado farms can also generate 0.66 MT of seed kernel and 0.06 MT of peel, a total of 0.72 MT of fresh biomass per hectare of avocado farms, with diverse (%DM) yields. The proportion of by‐products and (%DM) yields recorded from avocado and mango indicated statistical variation (*p* < 0.05) for both wet and dry seasons except for banana and avocado peel/seed, having similar values for all seasons.

**TABLE 5:** The total potential of fruit leftover biomass in wet and dry seasons of the study area.

Variables	Fruits and by‐products	Season		S.E.M.	*p* value
		Wet	Dry		
Total fresh biomass yield (t/ha)	Banana fruit	9.71^a^	6.83^b^	0.155	<0.001
	Discarded banana fruit	0.12^b^	0.18^a^	0.101	0.011
	Banana leaf	1.61^a^	1.37^b^	0.010	0.0236
	Banana pseudo stem	22.9^a^	18.8^b^	0.228	<0.001
	Banana peel	1.74	1.58	0.303	0.0745
	Mango fruit	2.80^b^	4.50^a^	0.011	<0.001
	Discarded mango fruit	0.62^b^	1.05^a^	0.036	<0.001
	Mango seed kernel	0.69^b^	1.18^a^	0.176	<0.001
	Avocado fruit	2.34^b^	3.33^a^	0.055	<0.001
	Avocado seed	0.55^b^	0.78^a^	0.046	<0.001
	Avocado peel	0.05	0.07	0.018	0.065
Per cent (%) of by‐products (wastes)/production size	Banana leaf biomass	4.47	4.76	0.455	0.365
	Discarded banana fruit	0.33^b^	0.59^a^	0.055	<0.001
	Banana pseudo stem	63.68^b^	65.39^a^	2.004	<0.001
	Banana peel	4.83	5.49	1.128	0.258
	Discarded mango fruit	22.14^b^	24.33^a^	1.139	0.024
	Mango seed kernel	24.67^b^	26.22^a^	1.112	0.031
	Avocado seed	23.5	23.4	1.138	0.233
	Avocado peel	2.14	2.40	0.491	0.652
DM yields (% t/ha)	Banana leaf	70.03^a^	59.59^b^	0.292	<0.001
	Discarded banana fruit	3.90^b^	5.52^a^	0.303	<0.001
	Banana pseudo stem	886.23^a^	727.56^b^	0.410	<0.001
	Banana peel	73.77^a^	66.99^b^	0.138	<0.001
	Mango seed kernel	34.10^b^	58.29^a^	0.711	<0.001
	Avocado seed	26.78	37.98^a^	2.185	<0.001
	Avocado peel	2.08	3.33	0.319	0.570

*Note*: Superscripts (a, b, c) across the same row show variation (*p* < 0.05) for each component.

Abbreviations: DM, dry matter; ha, hectare; S.E.M., standard error of mean; t, tonne.

### 3.7 Annual Potential of Tubers and Vegetable By‐Product Feed Resources

Major vegetables, including tuber crop production with leftover and by‐product potential, are indicated in Table 6. Among all sweet potato production contributes a higher (*p* < 0.05) amount of leaf and vein biomass, followed by potato peel, cassava leaf and peel, respectively. Among the vegetable by‐products, the higher DM yield (%) was obtained from sweet potato leaf and vein, followed by cassava peel, potato peel, cabbage leaf stripping, cassava leaf and discarded tomato biomass, respectively. Per cent of by‐products in relation to total production size indicated similarly with the higher (*p* < 0.05) share of sweet potato leaf and vein followed by potato peel, discarded tomato, cassava leaf, cassava peel and cabbage leaf stripping biomasses. However, except for cassava leaf and discarded tomato biomasses, all other vegetable by‐products were similar in both wet and dry seasons, respectively.

**TABLE 6:** The total potential of tubers and vegetable by‐products in wet and dry seasons of the study area.

Variables	Vegetable and by‐products	Season		S.E.M.	*p* value
		Wet	Dry		
Fresh biomass yield (t/ha)	Cassava tuber	20.5^b^	29.4^a^	0.103	<0.001
	Cassava peel	0.61^b^	0.84^a^	0.022	<0.001
	Cassava leaf biomass	0.74^a^	0.65^b^	0.024	<0.001
	Sweet potato tuber	22.20^b^	27.5^a^	0.261	<0.001
	Leaf and vein biomass	20.60^b^	24.80^a^	0.351	0.032
	Potato tuber	15.52^a^	13.52^b^	0.781	0.0350
	Potato peel	1.20	1.14	0.546	0.523
	Cabbage	56.80^a^	47.30^b^	1.352	0.036
	Discarded cabbage leaf	0.58	0.52	0.014	0.065
	Tomato fruit yield	6.25^b^	7.50^a^	0.131	<0.001
	Discarded tomato	0.46^a^	0.38^b^	0.013	<0.001
Per cent (%) of by‐products/production size	Potato peel	7.18	7.15	0.452	0.243
	S. potato leaf and vein	48.13	47.41	1.320	0.456
	Cassava peel	2.8	2.72	0.054	0.075
	Cassava leaf	3.38^b^	2.10^a^	0.472	0.017
	Discarded cabbage leaf	1.01	1.08	0.365	0.051
	Discarded tomato	6.85^a^	4.82^b^	0.421	0.033
DM yield (% t/ha)	S. potato leaf and vein	948.63^b^	1,142.04^a^	1.255	<0.001
	Potato peel	53.16	50.50	0.241	0.101
	Cassava peel	29.03^b^	39.98^a^	1.661	<0.001
	Cassava leaf	35.22^a^	26.52^b^	1.824	<0.001
	Discarded cabbage leaf	17.80	15.96	1.331	0.074
	Discarded tomato	7.72	6.38	0.055	0.363

*Note*: Superscripts (a, b, c) across the same row show variation (*p* < 0.05) for each component.

Abbreviations: ha, hectare; S.E.M., standard error of mean; t, tonne.

### 3.8 Seasonal Variations in the Use of Fruit By‐Products as Feed

Table 7 shows the use of fruit‐based feed resources specifically in an area where fruits were primarily cultivated and by‐products were readily available. The majority of respondents (91.8%, 94.6%, 29.7% and 100%, respectively) responded that leftover and damaged bananas, foliage (leaf and petiole), pseudo stems and banana peels are typically given to livestock. The Majority of these by‐products are provided to animals, particularly in dry seasons when alternative feed resources are limited. Additionally, 54.1% of respondents expressed their reaction to seed kernels and leftover mango that were used as feed. Offering avocado peels and seeds to calves as year‐round alternative feed was another practice mentioned by (94.6%) of respondents.

**TABLE 7:** Seasonal status and the use of fruit leftovers and by‐products as animal feed.

Fruit by‐products provided to your animals		Season of feeding (%)		Overall	*p* value
		Dry season	Wet season		
Discarded banana fruits (%)	Yes	81.1	10.7	91.8	
	No	4.1	4.1	8.2	<0.001
Banana foliage (%)	Yes	90.5	4.0	94.6	
	No	0.0	5.4	5.4	0.018
Banana pseudo stem (%)	Yes	24.3	5.4	29.7	
	No	10.7	59.6	70.3	0.026
Banana peel (%)	Yes	89.2	10.8	100	
	No	0.0	0.0	0.0	0.201
Mango fruits and by‐products (%)	Yes	47.9	6.2	54.1	
	No	10.8	35.1	45.9	<0.001
Avocado seed and by‐products (%)	Yes	82.4	12.2	94.6	
	No	0.0	5.4	5.4	0.006

### 3.9 Practical Utilization of Tubers and Vegetable Wastes With Seasons

Table 8 shows the trends in the use of vegetable and tuber by‐products as animal feed under various seasonal conditions throughout all areas. According to respondents’ responses, 59.4%, 78.4%, 88.5%, 100%, 37.8% and (91.8%) of respondents said that cassava leaf, cassava peel, potato peel, sweet potato leaf, vein and wasted tomato functionally contributed as livestock feed in both wet and dry seasons. Sweet potato leaf and vein are the most popular vegetable by‐products, followed by rejected potato peel, rejected cabbage leaf, cassava leaf and tomato biomass, respectively. All tuber and vegetable by‐products are widely used as livestock feed for the majority of respondents in both wet and dry seasons except cassava leaf and peel (*p* < 0.05) between seasons.

**TABLE 8:** Status of seasonal use of tuber and vegetable leftovers and by‐products as animal feed.

Tuber and veg. parts offered to animals		Seasons of feeding (%)		Overall	*p* value
		Dry season	Wet season		
Cassava leaf	Yes	54.4	5.0	59.4	<0.001
	No	5.8	35.2	40.6	
Cassava peel	Yes	70.3	8.1	78.4	0.025
	No	5.8	15.2	21.6	
Irish potato peel	Yes	43.4	45.1	88.5	0.145
	No	6.3	5.2	11.5	
Sweet potato leaf and vein	Yes	56.6	43.4	100	0.092
	No	0.0	0.0	0.0	
Discarded tomato	Yes	37.8	0.0	37.8	0.044
	No	3	59.2	62.2	
Cabbage leaf striping	Yes	36.1	55.7	91.8	0.147
	No	3.0	5.2	8.2	

*Note*: %, percentage of respondents.

Management aspects and modes of feeding practices for fruit by‐products are presented in Table 9. Among all, chopping and slicing is widely practised by the majority of respondents for all by‐products except banana peel. Moderate sun drying (wilting) of all fresh biomasses except discarded banana fruits was slightly practised by respondents who were used as feed for their livestock. Similarly, the mixing of fresh biomasses obtained from all fruit production is widely practised by respondents, and the majority of respondents provide fresh biomass as it is, except for banana pseudo stem due to their moisture content and physical appearance, which is not quite suitable for ingestion by livestock.

**TABLE 9:** Management and feeding practices of fruit by‐products reacted to by respondents (%).

	Preparation methods				
Biomasses	Slice	Dry	Mix	As it is	Overall (%)
Damaged banana	40.5	0.0	13.5	37.8	91.8
Banana foliage	70.2	5.4	13.5	5.4	94.6
Pseudo stem	21.6	2.7	5.4	0.0	29.7
Banana Peel	0.0	0.0	0.0	100	100
Mango flesh and seed	20.2	2.7	4.0	27.4	54.1
Avocado seed and peel	24.3	29.7	27.0	13.5	94.5

*Note*: %, percentage of respondents.

Modes of preparation and management practices for vegetable and tuber by‐products used as livestock feed are presented in Table 10. All management aspects and physical treatment methods and practices applied for vegetable by‐products to be suitable for livestock consumption for all components would also contribute to altering the practical utilization of tuber and vegetable by‐products.

**TABLE 10:** Mode of preparation for vegetable and tuber by‐products used as animal feed (%).

	Preparation methods				
Biomasses	Slice	Dry	Mix	As it is	Overall (%)
Cassava leaf	4.2	16.2	16.2	22.8	59.4
Cassava peel	2.6	20.2	18.0	37.8	78.4
Potato peel	9.2	9.8	28.6	40.9	88.5
Sweet potato leaf and vein	10.5	0.0	0.0	89.5	100
Cabbage leaf	4.0	1.6	14.7	71.5	91.8
Discarded tomato	0.0	6.7	10.1	27.0	37.8

*Note*: %, percentage of respondents.

On the basis of the reaction of respondents, chopping or slicing of all vegetable by‐products except rejected tomato biomass, drying or wilting commonly practised for all by‐products and mixing with other feed types would also be applied except for sweet potato leaves. The majority of respondents provided tubers and vegetable by‐products as they were, without applying any physical treatments.

## 4 Discussion

### 4.1 Demographic Characteristics of Respondents

Males made up the majority of respondent households, which was primarily due to the tradition of resource ownership status, as males are regarded as household heads in many parts of the country. The present study respondents were mostly between the ages of 36 and 45 years old. This indicates that the majority of responders are of productive age, which could be beneficial to livestock and agricultural output and development in general. In the area, the majority of respondents were illiterate (51.8%). According to focus group talks in all agro‐ecological zones, the higher degree of illiteracy was caused by a lack of awareness of education, and the area had less access to education in its early phases, but now all areas have equal access to primary‐level education. The low level of literacy demonstrates the necessity for adult education to improve education, as it affects the utilization of technology to increase agricultural productivity.

### 4.2 Land‐Holding Status of Respondents

The results of the current study for the landholding status of respondents were slightly lower than (2.5 ± 0.24), (2.03 ± 0.14) and (0.84 ± 0.24) for lowland, mid‐land and highland as compared to Burie districts (North‐Western Ethiopia), reported by Tegene and Belay (2019), but higher than (0.96 ha) and (0.98 ha) of the average farm size of Ethiopian farmers and Bahir Dar Zuriya districts reported by Headey et al. (2014) and Fentahun et al. (2020), respectively. The study result revealed that better private grazing and lands occupied by trees and shrubs were observed at highland and mid‐land agro‐ecologies than lowland agro‐ecologies. The focus group discussion substantiated that the majority of respondents do not have land for improved forage cultivation, and they do not have private grazing lands in lowland areas. This was reasoned as a shortage of land coupled with high population growth, competition for crop cultivation and the availability of some communal grazing lands in most parts of lowland districts.

### 4.3 Livestock Holding Status of Respondents

The current result of livestock holding status of smallholders in the area was lower than (5.86 ± 0.42) for cattle in the Ada'a and Sinana districts of central and Southeastern Ethiopia and Damot‐Gale district of Southern Ethiopia, reported by Sisay et al. (2021) and nearly in line with the report of Tahir et al. (2018) at the central highlands of mixed crop–livestock production systems. The facts behind the findings are variations in agro‐ecological zones that favour the existence of sheep and equines abundantly in the highlands, whereas goats survive at lowland altitudes in the area. Thus, the same proportion of cattle across all agro‐ecological zones might be attributed to feed scarcity; the contribution of natural pasture to livestock feed is generally declining due to the expansion of crop farming, particularly in lowland areas of the study districts.

It was also supported by a focus group discussion in lowland areas, as it was reflected that, currently, tomato, cabbage and onion have become emerging cash crops in addition to the banana and mango dominances of the area, particularly widely adopted at the lakeshores of Abaya Lake. Therefore, animal feed sourced from crop residue is declining as crop production turns from grain to fruit and vegetable production, particularly in lowland areas. Generally, production systems are categorized as grain–tubers–livestock at highlands, grain–fruits–livestock at mid‐land and fruit–grain–livestock at lowland areas, which is slightly different from the agricultural production systems of Gilgel Gibe catchment, Southwest Ethiopia, as reported by Duguma and Janssen (2021) and Hussen et al. (2016). Thus, it might be due to the availability of irrigation water on the lowlands, which favours the cultivation of different fruits as the main cash crop in the area.

### 4.4 Ranking Indices of Conventional Feed Resources, Fruits and Vegetable Wastes

In lowland areas, among non‐conventional feeds potentially used as alternative feed during dry seasons, banana by‐products and mango and avocado seeds with peels were widely used, but in mid‐ and highland areas, tubers and vegetable wastes with *Enset* by‐products, together with haulms and straws from legumes (haricot beans, field peas and beans), were considerably used and equally ranked 3rd–6th, respectively. Similarly, grain‐based straw at lowland, primarily obtained from maize‐producing farms and (teff, sorghum and barley straw) at mid‐land, and wheat straw at highland areas equally served as feed and ranked 2nd in all agro‐ecological zones. This could be due to agro‐ecological variation and environmental conditions that favour a variety of plants to be cultivated in mid‐ and highland areas. However, in lowland areas, due to the dominance of banana and mixed banana–mango production, leaf and other weeds and indigenous fodder trees are used to complement the dry season feed deficiency in the area.

Moreover, focus group discussions in lowland areas realized that grain‐based crop residues were declining over time due to the gradual transition of households from maize, cotton and haricot bean production to fruit and vegetable production. The availability of by‐products such as discarded mango seeds, avocado seeds and banana foliage, mainly mixed with atella (local alcohol by‐product), becomes prominent and usually provided to large and small ruminants in the area. The current finding related to the available feed resources prioritized by respondents was slightly different from the report of Hassan et al. (2020), where fodder trees, hay, crop residues, concentrates and stubble grazing were ranked 1st, 2nd, 3rd, 4th and 5th in Moyale districts of Southern Ethiopia. The reasons behind this variation could be attributed to the variation of mixed crop–livestock production systems of the current study area and dominant pastoral and agro‐pastoral production systems of Moyale districts in the region.

Natural pasture resources contribute throughout the year but are abundantly available during the main rainy seasons (May to September), which are the peak of crop cultivation seasons in the area. The current finding was in accordance with Tahir et al. (2018) and Metekia et al. (2021), who reported that the majority of crop residues are available during the dry season and the grazing land resources are available during long or short rainy seasons in the mixed crop–livestock production systems of the Ethiopian central highlands and the Kellem Wollega zone of Western Ethiopia. During dry seasons (November to April), both quantity and quality of available feed depleted and could not fully satiate the demand for livestock, as reported by Metekia et al. (2021) and Hassan et al. (2020) in Western and South‐Western Ethiopia, respectively.

### 4.5 Annual Production Potential for Fruit‐Based Feed Resources

Fruit farms generate a considerable amount of biomass that can be used as a livestock diet. The current finding was in contravention of the estimated biomass in terms of DM yield obtained from banana plants reported by Muhammad (2019) of Indonesia and nearly similar to Kramer (2014) for banana leaf and sheath from Hawaii. Moreover, the focus group discussion realized that some parts of banana pseudo stems remain on the farm until young sacker become strong, just to keep moisture and replenish some of the nutrients in the soil, in line with the report of Muhammad (2019). The result shows that, among the fruits cultivated and feed biomass yield in the area, banana plants pertained to the highest share, followed by mango and avocado fruits.

The by‐product amount recorded from avocado production of the current finding is different from (≥50%) of the total fruit size reported by Abraham et al. (2018) but nearly similar to Guzmán et al. (2013), who stated that avocado seed constituted nearly 10%–25% of the whole fruit biomass, which is in line with the current study finding (23.45%). The by‐products from these cash crops may only be available for certain periods and in specific seasons of the year, with a relatively large volume available during harvesting seasons. Under normal circumstances, large quantities of fruits and vegetables have been wasted during the harvesting season due to damage or poor grading for the market.

Some of these by‐products, rarely dumped at farm lands, might be served as compost, but many others are delivered to the local landfill, creating an additional environmental burden. Considerable values of these by‐products have been suggested by scholars, such as the incorporation of banana waste in ruminant diets by Muhammad (2019) and Ajebu et al. (2018) and the importance of avocado seed in ruminant diets by Talabi et al. (2016) and Gebremedhn and Alemu (2015). Similarly, banana leaves and pseudo stems can be used as supplementary feeds for animals that solely depend on natural pasture and crop residue diets, as reported by Adugna (2008).

Moreover, the efficiency of the utilization of available feed resources is significantly important, as feed scarcity affects the productivity of livestock. However, utilization efficiency was considered a great problem with the available biomasses, particularly banana pseudo stem and rejected mango fruit, which were not used as feed by (70.3%) and (45.9%) of the respondents, respectively. The finding was in accordance with Tegene and Belay (2019) and Metekia et al. (2021), in Western and Southern Ethiopia, respectively. Therefore, efficient utilization of available feed resources has to be taken into account in combination with knowledge of post‐harvest management, storage, conservation techniques, processing and improvement in quality.

### 4.6 Annual Production Potential for Vegetable and Tuber By‐Products

Tubers and vegetable farms would also generate valuable biomasses that can be used as livestock feed ingredients across agro‐ecological regions and seasons. Among tubers and vegetable by‐products, the biomass obtained from sweet potato production accounts for the highest share of biomass yield, DM% content and per cent of bio‐products per total production size, followed by potato peel, cassava leaf and peel biomasses. The current findings of DM yield for cabbage leaf striping are in accordance with Trinidad et al. (2019) and Nkosi et al. (2016) from South Africa and Spain, respectively. Moreover, tuber and vegetable by‐products (discarded cabbage leaf and potato peel) are reported as a good source of digestible nutrients for ruminants in developing countries (Ahmadi et al. 2020; Trinidad et al. 2019). The biomass yields of potato peel in the current study are in accordance with the report of Ncobela et al. (2017). The current finding of above‐ground biomass for sweet potato (*Ipomoea batatas*) is nearly similar to the reported values of Gabor et al. (2023). Moreover, the current biomass values of rejected tomato (0.42 t/ha and 5.83%) are lower than the findings of Abera (2020) from Shewa zone of Central Ethiopia. The current investigation prevailed that all the bulky vegetable and tuber crop by‐products, including discarded parts, have got much attention in maintaining farm animals feed requirements in the area.

### 4.7 Livestock Feeding Practices of Fruit and Vegetable By‐Products

On the basis of the reactions of respondents, fruit and vegetable processing residues have traditionally been used in animal nutrition as alternative feed ingredients. The majority of respondents have access to certain amounts and varieties of fruit and vegetable by‐products with different production potentials. The higher proportion of respondents used to feed fruit by‐products during the dry season, whereas few respondents were fed during the wet season, having varied with agro‐ecological zones (*p* < 0.05) for all by‐products except banana peel in the area. This implies that the biomass of fruit by‐products generated in each segment of value chains (production to consumption level) would be valuable in maintaining farm animals feed demand when the status of conventional feed resources declines.

The seasons of practical utilization of tubers and vegetable by‐products as livestock feed depend on the specific peak production and harvesting seasons. The majority of respondents reply that cassava leaf, peel and rejected tomato biomasses are widely used in the dry season with statistical variation (*p* < 0.05), whereas potato peel, sweet potato by‐products and cabbage leaf striping utilization practices were common irrespective of seasonal variations. This might be the attribution of available biomasses linked with the seasons of production, harvesting, trashing and consumption of vegetable products in the area. The trends of utilization of tomato leftovers (rejected tomatoes) are not widely branded as animal feed, but recently, ruminants have consumed them if they found them wherever accessible. However, study findings of Gebeyew et al. (2015) suggested that tomato pomace has the potential to replace 100% of the concentrate mixture to supplement sheep fed on hay as basal diet. Generally, available by‐products are used to maintain feed shortage problems, especially during dry seasons when other feed resources are scarce. Therefore, fruit and vegetable by‐products become vital for sustainability and complementing feed scarcity during dry season.

## 5 Conclusion

The study discovered the potential of fruits and vegetable by‐products as alternative feed sources for livestock based on respondents’ practices and field observation. Furthermore, the study found that seasonal and agro‐ecological zone variations contributed to differences and variability of key feed resources, livestock holding status and land resources. The study also indicates a significant and huge amount of lost and wasted fruits and vegetables and their components, which represent not only losses of edible food materials but also wasting of by‐products, including bioactive compounds, including nutrient ingredients. On the basis of estimation and recorded values of feed resources generated from fruit farms, 24.14 MT of banana by‐products, 1.76 MT of mango by‐products and 0.72 MT of avocado by‐products of fresh biomass were obtained from a hectare of banana, mango and avocado farms with different DM yields, respectively. Similarly, 22.7 MT of sweet potato leaf and vein, 1.41 cassava metric tonnes of peels and leaf, 1.12 MT of potato peels, 0.55 MT of cabbage leaf and 0.42 MT of discarded tomato fresh biomass were obtained from a hectare of land with different DM yields, respectively. Because the demand and supply of feed resources mismatched, if persisted with conventional feed resources alone, livestock production is expected to decline. Considerable attention should be given to fruit and vegetable wastes as an alternative measure and enhancing efficiency of utilization for sustainability and to mitigate the existing farm animal feed shortage in the area. Thus, livestock feed production using fruit and vegetable by‐products is an eco‐friendly, sustainable and efficient waste management option. There is also a need to utilize more novel techniques, like conservation and processing techniques, with respect to waste materials to achieve higher retrieval rates of bioactive compounds.

## Author Contributions

Mitiku Yohannes conceptualized the idea of the study, curated the data, performed formal analysis and investigation, designed methodology, administered the project and wrote the original draft. Yisehak Kechero curated the data, performed formal analysis and investigation, designed methodology, administered the project and performed data analysis. Yilkal Tadele performed a review, performed formal analysis, compiled the report and drafted the manuscript.

## Ethics Statement

The authors confirm that they adhered to the ethical rules of the journal, as outlined on the author guidelines page. However, the study did not use animals for direct experimentation but rather field observation, field measurement, and laboratory analysis for dry matter.

## Conflicts of Interest

The authors declare no conflicts of interest.

### Peer Review

The peer review history for this article is available at https://www.webofscience.com/api/gateway/wos/peer‐review/10.1002/vms3.70349.

## Data Availability

Upon reasonable request, the corresponding author can provide the data supporting all of the study's findings.
